# Getting the best GRIP on blood pressure control: Investigating a cost-effective isometric handgrip alternative

**DOI:** 10.1177/17423953211049753

**Published:** 2021-11-06

**Authors:** Jared J Richards, Paula M van Wyk, Cayla N Wood, Logan P Shea, Ian Swaine, Phillip Levy, Jamie Crawley, Kevin J Milne, Cheri LM McGowan

**Affiliations:** 1 Department of Kinesiology, Faculty of Human Kinetics, 8637 University of Windsor, Windsor, Canada; 2 Department of Life & Sport Sciences, 4918 University of Greenwich, Medway Campus, London, UK; 3 Department of Emergency Medicine, School of Medicine, 12267 Wayne State University, Detroit, USA; 4 Department of Nursing, Faculty of Nursing, 8637 University of Windsor, Windsor, Canada

**Keywords:** Cardiovascular disease, hypertension, blood pressure, isometric, exercise, treatment

## Abstract

**Objectives:**

The World Health Organization emphasises the need for cost-effective alternative methods to lower blood pressure (BP). Endorsed nationally in HTN guidelines, isometric handgrip (IHG) training is an alternative method of BP control. The purpose of this study was to compare the BP, heart rate (HR) and rates of perceived exertion (RPE) responses between a bout of IHG training performed using the traditional computerized device and a more affordable, inflatable stress ball.

**Methods:**

Twenty healthy adults performed one bout (4, 2-min isometric contractions, with 1-min rests between each contraction at 30% maximal voluntary contraction) of IHG training using the traditional computerized device, and one bout with the inflatable stress ball. BP, HR, and RPE were recorded.

**Results:**

No statistically significant differences between devices were observed with HR, BP, and RPE (*p* < 0.05). However, average RPE for both devices ranged between 5 and 6 indicating that participants were rating 30% of their MVC, consistent with previous work.

**Discussion:**

The similar cardiovascular and psychophysical responses provide support for the potential use of this low individual- and provider-burden, cost-efficient IHG device, and lay the foundation for a future training study to test the hypothesis of benefit.

## Introduction

Cardiovascular disease (CVD) is the number one cause of death globally.^1^ In 2016, more than 17 million or 31% of all global deaths were attributed to CVD.^1^ Hypertension (HTN; historically defined as resting blood pressure (BP) ≥140/90 mmHg)^2–5^ is the leading cause of CVD and CVD-related mortality, and the number one modifiable risk factor for this disease.^6–9^ Affecting more than 1 billion people worldwide,^10^ the World Health Organization (WHO) has identified HTN as a global epidemic.^11^

Cornerstone recommendations for prevention, treatment, and management of HTN include a reduction in alcohol consumption, cessation of tobacco smoking, maintenance of a healthy body weight, improvements in diet, and increases in physical exercise (e.g. aerobic exercise combined with dynamic resistance training) in addition to routine activities of daily living.^3,4^ When lifestyle modifications do not successfully control BP to within clinical target ranges,^12^ pharmacotherapy is used concomitantly.^3,4,13,14^ Despite these intervention strategies, less than 50% of individuals with HTN have their condition controlled to within clinical targets.^15^ This may be attributable to many factors, including non-adherence to medication, diet and/or physical activity regimens, and the cost of treatment.^16,17^ It is crucial to develop complementary and cost-effective strategies that can be used alone or in conjunction with traditional BP-lowering interventions to better control BP.^1^

Alternative strategies have been investigated with varying degrees of success (e.g. meditation, biofeedback, device-guided breathing), including isometric handgrip (IHG) training.^16,17^ After decades of accumulating proof-of-concept evidence, the American Heart Association and American College of Cardiology (AHA/ACC) now collectively endorse IHG training as a treatment for HTN management in their most recent guidelines.^4,16^ Typically, an IHG protocol involves the use of a computerized handgrip dynamometer with participants performing 4, 2-min sustained squeezes (isometric contractions), each separated by 1-min rest intervals,^18^ at 30% of an individual's maximum voluntary contraction (MVC), performed 3–5 times per week for 8–10 weeks.^16,17,19,20^ However, there is concern regarding the accessible and economic feasibility of using such devices for widespread implementation.

The high cost of the computerized IHG dynamometer (upwards of $600 USD^21^^)^ and lack of insurance coverage for the device creates an economic barrier to its widespread uptake and implementation.^1,17,22,23^ This notion of using an alternative and affordable device for IHG is supported by prior studies showing short-term training-induced reductions in BP for normotensive older adults.^22^ However, most alternative methods investigated to date present barriers limiting their use – whether it be expensive hand or leg dynamometer devices, difficult to calibrate spring-loaded IHG devices, or challenging wall squats.^22,24,25^

An alternative cost-efficient device not yet explored is the widely known “stress ball”. This device is not reliant on calibration equipment to regulate force and can be performed by individuals with barriers preventing exercise involving lower extremities. Recent work by Morrin et al.^26^ lay a foundation for such investigation as they determined that a rating of perceived exertion (CR-10; RPE) of 6 out of 10 during a 2-min IHG contraction is equivalent to 30% MVC.^26^ To date, no study has investigated the acute (during a bout) or chronic (training) effects of IHG on BP or other indices of cardiovascular and psychophysical function implemented when using a stress ball.

Before a long-term training investigation can be undertaken, it is important to first examine and compare the acute stimuli of the (inflatable) stress ball with the computerized dynamometer, including cardiovascular (BP and heart rate, HR), and RPE (similar to Morrin et al.)^26^ responses. Therefore, the primary objective of the present investigation was to test the hypothesis that an acute bout of IHG utilizing an inexpensive, readily available stress ball would elicit similar cardiovascular responses as the traditional computerized dynamometer. In addition, it was anticipated that an acute bout of IHG would elicit a similar RPE (of approximately 6 on the CR-10) using both devices.

## Methods

### Study participants

G*power calculations were conducted using within factors and within-between factors for a repeated measures test It was determined that the required minimum number of participants needed to complete the isometric handgrip session for two devices was 18, with (1- β error probability; power = 0.95). Thus, twenty healthy adults (≥18 years old, resting BP < 135/85 mmHg, no overt disease, non-smoking, no pharmacological treatments present in their daily routines, with the exception of birth control pills, were recruited from within southwestern Ontario. Recruitment involved posters, public announcements, and emails. All participants provided written and informed consent, and all procedures were cleared by an institutional research ethics board (REB #17-069).

### Study design

Following consent and determination of eligibility, participants completed a familiarization and testing session. Each of these sessions involved the measurement of HR and BP while performing a bout of IHG using: (i) a computerized IHG dynamometer (Zona Series 2, Zona Health, Boise, ID, USA) and, (ii) a store-bought (3 inches in diameter, inflatable, with standard sport valve) stress ball (AllBall, Sportime, Thailand). The order of the two IHG methods (i.e. computerized IHG dynamometer and stress ball) were randomized for each participant with a 30-min stabilization period between each bout. Total time to complete the collective procedures was approximately 3 h and included 2 points of contact (i.e. visit 1 and visit 2).

### Visit 1: eligibility and familiarization

Following informed consent, eligible participants completed the Physical Activity and Readiness Questionnaire plus (PAR-Q+) and a medical questionnaire with the intent to screen for any ailments that may exclude them from participating. Resting BP was measured after 10-min of seated rest to ensure inclusion BP criterion was met (<135/85 mmHg) according to standard protocol^18,27,28^ (Dinamap Carescape v100, Critikon 23–33 cm cuff, Tampa, Florida, USA). Four measurements were acquired, with 2-min rest periods between each measurement. The last 3 BP values were averaged and used in the final analysis as the first measure can be overestimated.^18,27,28^ Participants meeting all eligibility criteria proceeded to protocol familiarization.

During the familiarization session, participants practiced testing day procedures. The session began with determination of a maximal voluntary contraction (MVC) on each device. Order of device was randomized, but training always began using a device in the right hand followed by the second device in the left hand. The MVC for the computerized dynamometer was automatically calculated from internal linear load cells. The MVC for the stress ball was calculated using a digital air pressure gauge. Subsequently, respective 30% MVC's were calculated; for the computerized IHG dynamometer this calculation was done automatically, and for the stress ball by calculating 30% of maximum pounds per square inch (PSI) attained during the MVC.

Once completed, the participant performed a 2-min IHG bout at 30% MVC using their right hand. After the bout was finished, the participant was given the chance to review the CR-10 scale, provide a rating based on the scale, and have their BP measured as per testing day procedures. After a 1-to-2-min rest period, this process was completed on the left hand and the alternative device was used for the second bout to ensure minimal fatigue experienced during this familiarization session.

### Visit 2: testing day

After confirmation of ongoing consent, and at least 24 h after the familiarization session, a single testing session occurred. All participants were tested in a temperature-controlled room (within normal room temperature range^18^^)^ in the morning to control for the effects of circadian rhythm on BP. Participants were asked to refrain from vigorous physical activity for the previous 24 h, and they were tested 2 h postprandial and at least 12 h post-caffeine consumption. To minimize the effects of a full bladder on BP, participants were asked to void their bladder prior to testing.

Participants were seated with feet uncrossed and flat on the floor for the duration of the testing period, with their right and left forearms resting on a table in front of them at an approximate 90-degree angle. Participants were outfitted with the necessary equipment to assess BP (as described above) and HR. Baseline BP was measured following 10 min of seated rest The protocols were separated by a 30-min rest period (minimum) or until the participant's BP had returned to near baseline values.

**Figure 1.**
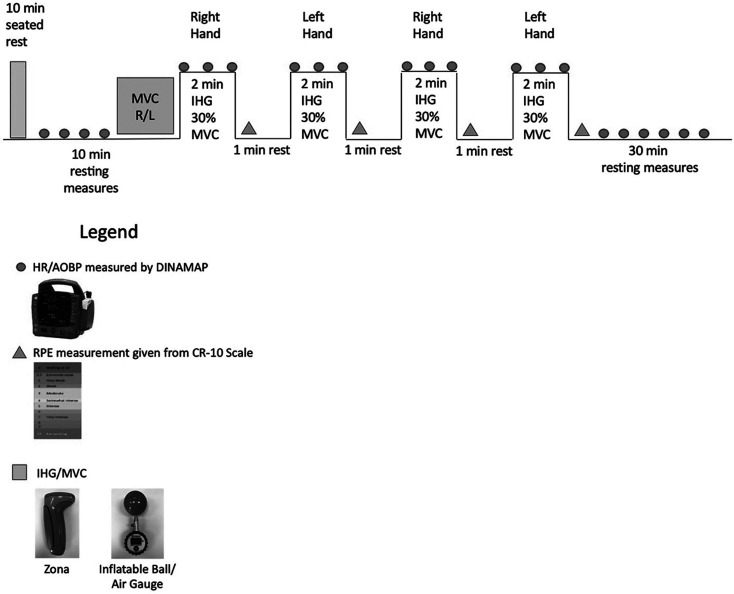
Testing protocol note: The first rectangle on the left is the end of seated rest, each circle is a blood pressure (BP) and heart rate (HR) measurement completed with automated office blood pressure measurements (AOBP) via dinamap. The protocol began at the square with a maximum voluntary contraction (MVC), where 30% was calculated for both the right (R) and left (L) hands. After the participant engaged in 4, 2-min bilateral isometric handgrip (IHG) contractions, a subjective rating of perceived exertion (RPE) was recorded after each contraction represented by the triangles. A 30-min stabilization period (with BP and HR measurements) concluded each IHG bout. Once 30 min was completed the protocol began at the MVC square again with the second device.

In both conditions, the IHG bout consisted of 4, 2-min bilateral (i.e. right and left hand) contractions at 30% MVC each separated by a 1-min rest period, where BP and HR were measured every minute and RPE was recorded after each contraction (see Figure 1)

### Statistical analysis

Data were analyzed using IBM SPSS Statistics 23 software (SPSS Inc., Chicago, Illinois, USA) and statistical significance was determined at *P* ≤ 0.05. Two-way repeated measures analysis of covariance (RMANCOVA) were used to determine the effects of IHG device (independent variables: computerized or stress ball) on BP (systolic blood pressure, SBP, and diastolic blood pressure, DBP), HR, and RPE scores (dependent variables). To determine specific differences between means, a Bonferroni *post-hoc* test was employed where appropriate. Data are presented as means and standard deviations unless otherwise noted. Additionally, assumptions of sphericity were met for all conditions, with the exception of DBP, where the assumption for the two-way interaction between device and time was violated (x^2^(2) = 11.31, *P* = 0.05). Thus, Greenhouse-Gieser was employed (€ = 0.70).

## Results

Twenty participants met the eligibility criteria and were enrolled in the study (Table 1). All participants completed visit 1 and visit 2, and adhered to pre-testing instructions.

**Table 1.** Participant characteristics.

Variable	Baseline value
n (% women)	20 (50%)
Age (years)	24.70 ± 5.13
Mass (kg)	74.16 ± 18.15
Height (cm)	171.67 ± 12.43
Resting SBP (mmHg)	107.93 ± 16.14
Resting DBP (mmHg)	58.68 ± 6.77
Resting HR (bpm)	66.01 ± 8.61

Note: DBP: diastolic blood pressure; HR: heart rate; SBP: systolic blood pressure.

Values are x̄ ± SD.

### Comparison of blood pressure and heart rate

Statistically significant BP and HR differences between devices were revealed through the RMANCOVA, whereby SBP and DBP were higher when the IHG protocol was performed using the stress ball, yet there was a greater HR response to the computerized IHG protocol (*P* ≤ 0.05). Importantly, these differences were not present upon pairwise comparison analyses (see Table 2). The details of the analyses are as follows:

**Table 2.** Cardiovascular effects.

Variable	Computerized device	Stress ball
SBP (mmHg)	8.23 ± 9.47	9.10 ± 7.36
DBP (mmHg)	7.13 ± 7.77	8.86 ± 9.90
HR (bpm)	6.98 ± 7.50	6.53 ± 8.20

Note: DBP: change in diastolic blood pressure; HR: change in heart rate; SBP: change in systolic blood pressure. Values are x̄ ± SD.

With respect to SBP, based on multivariate analysis there were interaction effects for device and time (*F* (3,48) = 4.52, *P* = 0.007); device and order (*F* (1,16) = 5.06, *P* = 0.04); and a three-way interaction between device, time and order (*F* (3,48) = 2.84, *P* = 0.05). As noted above, after pairwise comparisons, statistically significant differences for SBP between devices were no longer observed (*P* = 0.62). However, men had a significantly higher SBP response throughout the IHG protocols when compared to women (10.92 ± 1.13 mmHg for men and 7.40 ± 1.01 mmHg for women).

There were no statistically significant interactions observed with respect to DBP. However, there were within-subject main effects for both devices (*F* (1,16) = 5.11, *P* = 0.04), and time (*F* (3,48) = 3.29, *P* = 0.03). Like SBP, pairwise comparison of each device denoted that although the stress ball elicited a higher DBP throughout the protocol, this was not statistically significant from the computerized IHG dynamometer (*P* = 0.35).

A statistically significant interaction was observed between device and order (*F* (1,16) = 22.91, *P* < 0.001) with respect to HR multivariate analysis. However, this was no longer significant upon further analyses of between-subject effects for order (*P* = 0.64) and device (*P* = 0.73).

### Comparison of subjective rating of perceived exertion

An interaction between time and sex (*F* (3,48) = 2.83, *P* = 0.048) for RPE, and main effects for time (*F* (3,48) = 4.81, *P* = 0.005), fitness (*F* (1,16) = 5.062, *P* = 0.04) were noted, but no main effects were observed for sex, order and device regarding RPE. Although fitness was initially significant based on between-subject interactions (*F* (1,16) = 5.062, *P* = 0.039), upon analysis of pairwise comparisons this did not remain significant.

Pairwise comparison of devices displayed the computerized device to be perceived harder by 0.337 (CI 95%, −0.27 to 0.94), but this result was also not statistically significant (*F* (1,16) = 0.51, *P* = 0.26). In contrast, pairwise comparison of contractions revealed some statistically significant differences, but these differences were not meaningful (see Table 3).

**Table 3.** Subjective rating of perceived exertion.

Scale	Device	Cont. 1	Cont. 2	Cont. 3	Cont. 4
RPE (1-10)	Computerized	5.25 ± 1.80^†‡^	5.60 ± 1.79^†‡^	6.05 ± 1.93*^+^	6.15 ± 1.79*^+^
RPE (1-10)	Stress Ball	5.10 ± 1.59^†‡^	5.00 ± 1.69^†‡^	5.70 ± 1.78*^+^	5.90 ± 2.02*^+^

Note: Rating of perceived exertion (RPE). Values are mean ± standard deviation (x̄ ± SD).

*Significant differences from contraction 1, ^+^Significant differences from contraction 2, ^†^Significant differences from contraction 3, ^‡^Significant differences from contraction 4.

## Discussion

The long-term effects of IHG training on BP are well documented.^20^ However, widespread uptake and continued participation in this form of exercise training could have been hindered by the barrier of accessibility, including cost, for the computerized dynamometer. This study makes the following important contributions to the literature: (i) the first evidence that a cost-effective alternative can replicate acute HR and BP responses elicited by more expensive and traditional IHG devices, laying a foundation for future training studies, and (ii) provide novel data on the acute effects of IHG on hemodynamic response, and concomitant RPE.

Based on the data analysis, there were no statistical differences observed between each device based on the examination of SBP, DBP, and HR. These results support the viability of IHG using stress balls as a potential cost-effective, readily accessible treatment opportunity for lowering BP worldwide.^1,11^ The use of a simple inexpensive analogue pressure indicator, inserted into the air valve of the ball, allowed the exercise intensity to be regulated according to a pre-calculated target (%MVC). This method of regulating isometric exercise intensity has provided consistent findings in relation to repeatable reductions in resting BP after training. However, the novel stress ball, used in the current study, did not allow data to be recorded and handled electronically, in the same way that is possible when using the computerized device. Therefore, future studies are needed to explore the long-term efficacy of such simple, inexpensive devices, when used to investigate the effects of IHG training on resting BP.

The RPE data is important with respect to insight into the potential for widespread implementation and uptake of IHG training as a BP management treatment. The current study provides support for the long-term potential to use RPE to self-regulate isometric exercise intensity, because both devices elicited a similar level of perceived exertion, which was low (RPE level of 5–6). Building on this was the observation that 30% MVC equated to an RPE of 5–6 for each contraction, a finding similar to the work of Morrin et al.^26^ In that study, when participants were asked to self-regulate their exercise intensity according to CR-10 level ‘6’, the ‘produced’ intensity (%MVC) varied across the 4, 2-min periods of exercise. This work revealed that the isometric exercise intensity started at approximately 43% MVC, in the first 30 s and fell to approximately 23%MVC in the last 30 s, when participants attempted to produce (self-regulate) exercise at an intensity of CR-10 level ‘6’. The difference between the current study and the experiment by Morrin et al.,^26^ was that participants rated the IHG exercise the same organically, without previous practice on what 30% of their MVC “felt like”.

However, the present study involved ‘estimation’ of exertion (RPE) and, in order to self-regulate isometric exercise intensity during training, it is integral to learn how to ‘produce’ an exercise intensity, according to their perception of exertion. Production of exercise intensity requires exertion regulation ‘learning’. Soriano-Maldonado et al.,^29^ found that a ‘learning protocol’ improved the validity of RPE to self-regulate exercise intensity during indoor cycling. In the present study, where exercise intensity was not self-regulated, but regulated by participant adherence to a ‘target’ (‘squeezing pressure’ to calculated 30%), RPE scores across 4 periods of IHG exercise remained fairly constant (5.1 to 5.9 during stress ball exercise).

The current study provides insight into the ability of the participant to self-regulate IHG exercise without the feedback from the computerized device. A prominent limiting factor of the device investigated in the seminal work of Millar et al.^22^ was that trained personnel were required to regulate training intensity. The possibility that an individual can use the stress ball, self-regulating 30% based on the CR-10 scale (rating of approximately 6) without trained personnel, could overcome some barriers relating to the accessibility of IHG training, in socially-deprived groups within various populations. However, given the challenge of accurate self-regulation, further study is required prior to wide-spread applicability.

The authors recognize the results observed from the testing population of healthy, young, normotensives possess limitations, which could potentially have different results when compared to unmedicated hypertensives or medicated hypertensives under the same testing conditions. However, the effectiveness of IHG in both populations has been well documented.^4,13,17–20,30–33^ Additionally, data that is recorded and handled electronically, when performing IHG on a computerized device, has the potential to provide increased accuracy as opposed to manual assessment with the inflatable stress ball. Despite this, the results from this study may be translatable and would support future studies with hypertensive populations and improvement of device quality.

## Clinical significance

The demonstration of similar cardiovascular and RPE responses to a bout of IHG using a computerized dynamometer and an inexpensive stress ball suggests that IHG training performed using the latter may have similar BP-lowering benefit. Further, gaining a better understanding of the subjective RPE will address a gap exposed by previous investigators^26^ and generate knowledge that will inform programing decisions as to the feasibility of this device as a standard of care treatment. This project lays the foundation for future studies to test efficacy and outcome benefit, further promoting IHG as a global treatment option for BP management.

Importantly, this work aligns with the key health priorities of the WHO, which emphasize enhanced feasibility, low maintenance, and inexpensive ways to effectively prevent, treat, and manage HTN.^1^ Our findings may be of a particular relevance to individuals residing in low-to-middle income countries (LMIC) where HTN rates contribute to 75% of the global disease burden.^1^ Healthcare system-, provider- and/or patient-level barriers, such as lack of access to care, high provider burden, poor healthcare staffing, low patient health literacy, and lack of treatment compliance, are also contributing factors.^24,29^^,34^ Taken together, implementation of cost-effective, readily available BP-lowering treatments that work, have a low provider and patient burden, and offer a high potential for uptake and long-term continuation are urgently needed in LMICs. The potential to offer IHG training using a stress ball as a BP-lowering standard of care treatment in primary care is innovative and timely.

## Conclusions

The current study provided evidence for similar hemodynamic (HR and BP) and psychophysical (RPE) responses to a bout of IHG using a traditional computerized dynamometer and a stress ball. These findings support the need for future studies to test efficacy and outcome benefit using a more scalable approach, further promoting IHG as a treatment option for BP management.

## References

[1] World Health Organization (WHO). Cardiovascular diseases (CVDs). https://www.who.int/news-room/fact-sheets/detail/cardiovascular-diseases-(cvds) (accessed 15 January 2019).

[2] Gee ME Campbell N Sarrafzadegan N, et al. Standards for the uniform reporting of hypertension in adults using population survey data: recommendations from the world hypertension league expert committee. J Clin Hypertens 2014; 16: 773–781. 10.1111/jch.12387 PMC8031637 25157607

[3] Nerenberg KA Zarnke KB Leung AA, et al. Hypertension Canada’s 2018 guidelines for diagnosis, risk assessment, prevention, and treatment of hypertension in adults and children. Can J Cardiol 2018; 34: 506–525. 29731013 10.1016/j.cjca.2018.02.022

[4] Whelton PK Carey RM Aronow WS, et al. 2017 ACC/AHA/AAPA/ABC/ACPM/AGS/APhA/ASH/ASPC/NMA/PCNA guideline for the prevention, detection, evaluation, and management of high blood pressure in adults. JACC 2018; 71: 127–248.

[5] Williams B Mancia G Spiering W, et al. 2018 ESC/ESH guidelines for the management of arterial hypertension. Eur Heart J 2018; 39: 3021–3104. 30165516 10.1093/eurheartj/ehy339

[6] Ezzati M Lopez A Rodgers A, et al. Selected major risk factors and global and regional burden of disease. Lancet 2002; 360: 1347–1360. 12423980 10.1016/S0140-6736(02)11403-6

[7] Danaei G Ding EL Mozaffarian D, et al. Correction: the preventable causes of death in the United States: comparative risk assessment of dietary, lifestyle, and metabolic risk factors. PLoS Med 2011; 8: 10–1371. 10.1371/journal.pmed.1000058 PMC2667673 19399161

[8] Joffres M Falaschetti E Gillespie C, et al. Hypertension prevalence, awareness, treatment and control in national surveys from England, the USA and Canada, and correlation with stroke and ischaemic heart disease mortality: a cross-sectional study. BMJ Open 2013; 3: e003423. 10.1136/bmjopen-2013-003423 PMC3758966 23996822

[9] Benjamin E Blaha M Chiuve S, et al. Heart disease and stroke statistics - 2017 update: a report from the American heart association. Circulation 2017; 135: 146–603. 10.1161/CIR.0000000000000485 PMC5408160 28122885

[10] Fisher ND Curfman G. Hypertension—A public health challenge of global proportions. Jama 2018; 320: 1757–1759. 30398584 10.1001/jama.2018.16760

[11] World Health Organization (WHO). A global brief on hypertension. http://apps.who.int/iris/bitstream/handle/10665/79059/WHO_DCO_WHD_2013.2_eng.pdf;jsessionid=5139C97E80CFA29746119E43C916BB65?sequence=1(accessed 15 January 2019).

[12] Eckel RH Jakicic JM Ard JD, et al. 2013 AHA/ACC guideline on lifestyle management to reduce cardiovascular risk: a report of the American college of cardiology/American heart association task force on practice guidelines. JACC 2014; 63: 2960–2984. 24239922 10.1016/j.jacc.2013.11.003

[13] Owen A Wiles J Swaine I. Effect of isometric exercise on resting blood pressure: a meta-analysis. J Hum Hypertens 2010; 24: 796–800. 20182455 10.1038/jhh.2010.13

[14] Leung AA Daskalopoulou SS Dasgupta K, et al. Hypertension Canada’s 2017 guidelines for diagnosis, risk assessment, prevention, and treatment of hypertension in adults. Can J Cardiol 2017; 33: 557–576. 28449828 10.1016/j.cjca.2017.03.005

[15] Go AS Mozaffarian D Roger VL. Heart disease and stroke statistics-2013 update. Circulation 2013; 127: 143–152. 23283859 10.1161/CIR.0b013e318282ab8f

[16] Brook RD Appel LJ Rubenfire M, et al. Beyond medications and diet: alternative approaches to lowering blood pressure. A scientific statement from the American heart association. Hypertension 2013; 61: 1360–1383. 23608661 10.1161/HYP.0b013e318293645f

[17] McGowan CL Proctor DN Swaine I, et al. Isometric handgrip as an adjunct for blood pressure control: a primer for clinicians. Curr Hypertens Rep 2017; 19: 51. 28528376 10.1007/s11906-017-0748-8

[18] Badrov MB Bartol CL DiBartolomeo MA, et al. Effects of isometric handgrip training dose on resting blood pressure and resistance vessel endothelial function in normotensive women. Eur J Appl Physiol 2013; 113: 2091–2100. 23588257 10.1007/s00421-013-2644-5

[19] Millar PJ McGowan CL Cornelissen VA, et al. Evidence for the role of isometric exercise training in reducing blood pressure: potential mechanisms and future directions. Sports Med 2014; 44: 345–356. 24174307 10.1007/s40279-013-0118-x

[20] Inder JD Carlson DJ Dieberg G, et al. Isometric exercise training for blood pressure management: a systematic review and meta-analysis to optimize benefit. Hypertension Res 2016; 39: 88–94. 10.1038/hr.2015.111 26467494

[21] Zona Health. FAQ. https://zonahealth.ca/pages/faq?locale=en (accessed 16 January 2019).

[22] Millar PJ Bray SR MacDonald MJ, et al. The hypotensive effects of isometric handgrip training using an inexpensive spring handgrip training device. J Cardiopulm Rehabil Prev 2008; 28: 203–207. 18496321 10.1097/01.HCR.0000320073.66223.a7

[23] Zhang M Fang XX Li ME, et al. Handgrip exercise elevates basilic venous hemodynamic parameters in healthy subjects. Int J of Nurs Sci 2014; 1: 389–393.

[24] Wiles JD Goldring N Coleman D. Home-based isometric exercise training induced reductions resting blood pressure. Eur J Appl Physiol 2017; 117: 83–93. 27853886 10.1007/s00421-016-3501-0

[25] Baross AW Wiles JD Swaine IL. Effects of the intensity of leg isometric training on the vasculature of trained and untrained limbs and resting blood pressure in middle-aged men. Int J Vasc Med 2012; 2012: 964697. DOI: 10.1155/2012/964697. PMC3443998 22991668

[26] Morrin NM Stone MR Swaine IL, et al. The use of the CR-10 scale to allow self-regulation of isometric exercise intensity in pre-hypertensive and hypertensive participants. Eur J Appl Physiol 2018; 118: 339–347. 29214462 10.1007/s00421-017-3774-y

[27] Pickering TG Hall JE Appel LJ, et al. Recommendations for blood pressure measurement in humans and experimental animals part 1: blood pressure measurement for humans: a statement for professionals from the subcommittee of professional and public education of the American heart association council on high blood pressure research. J Clin Hypertens 2005; 45: 142–161. 10.1161/01.HYP.0000150859.47929.8e 15611362

[28] Somani YB Baross AW Brook RD, et al. Acute response to a 2-min isometric exercise test predicts the blood pressure-lowering efficacy of isometric resistance training in young adults. Am J Hypertens 2017; 31: 362–368. 10.1093/ajh/hpx173 29036548

[29] Soriano-Maldonado A Romero L Femia P, et al. A learning protocol improves the validity of the borg 6–20 RPE scale during indoor cycling. Int J Sports Med 2014; 35: 379–384. 24165960 10.1055/s-0033-1353166

[30] Musinguzi G Bastiaens H Wanyenze RK, et al. Capacity of health facilities to manage hypertension in Mukono and Buikwe districts in Uganda: challenges and recommendations. PloS One 2015; 10: e0142312. 26560131 10.1371/journal.pone.0142312 PMC4641641

[31] Araújo CGSD Duarte CV Gonçalves FDA, et al. Hemodynamic responses to an training protocol. Arq Bras Cardiol 2011; 97: 413–419. 22011802 10.1590/s0066-782x2011005000102

[32] Olher RDRV Bocalini DS Bacurau RF, et al. Isometric handgrip does not elicit cardiovascular overload or post-exercise hypotension in hypertensive older women. Clin Interv Aging 2013; 8: 649–655. 23766641 10.2147/CIA.S40560 PMC3678710

[33] Carlson DJ Inder J Palanisamy SK, et al. The efficacy of isometric resistance training utilizing handgrip exercise for blood pressure management: a randomized trial. Medicine (Baltimore). 2016; 95: e5791. 28033302 10.1097/MD.0000000000005791 PMC5207598

[34] Mills KT Bundy JD Kelly TN, et al. Global disparities of hypertension prevalence and control: a systematic analysis of population-based studies from 90 countries. Circulation 2015; 134: 441–450. 10.1161/CIRCULATIONAHA.115.018912 PMC4979614 27502908

